# Short Vestibular and Cognitive Training Improves Oral Reading Fluency in Children with Dyslexia

**DOI:** 10.3390/brainsci11111440

**Published:** 2021-10-29

**Authors:** Simona Caldani, Lionel Moiroud, Carole Miquel, Vanessa Peiffer, Alessandro Florian, Maria Pia Bucci

**Affiliations:** 1 UMR 7114 MoDyCo, CNRS Paris University, 92001 Nanterre, France; simona.caldani@gmial.com (S.C.); lionel.moiroud@gmail.com (L.M.); 2 Vestibular and Oculomotor Evaluation Unit, ENT Department, Robert Debré University Hospital, 75019 Paris, France; 3 Pôle Santé Henri Arnaud, 81370 Saint-Sulpice-la-Pointe, France; carolemiquel@hotmail.com (C.M.); vanessapeiffer@hotmail.fr (V.P.); 4 BeonSolution Society, 31059 Trevise, Italy; beonsolutions@gmail.com

**Keywords:** children, dyslexia, vestibular and cognitive training, reading speed

## Abstract

(1) Background: This study explored the effect of short vestibular and cognitive training on the reading speed in dyslexic children. (2) Methods: The reading speed was evaluated by using a reading test (Évaluation de la Lecture en FluencE, ELFE) in a crossover design before (baseline) and after vestibular training (post VT) and no vestibular training (post no VT). Nineteen dyslexic children (9.48 ± 0.15 years) participated in the study. The vestibular and cognitive training (software developed by BeonSolution S.r.l.) consisted in four exercises presented on a Wacom tablet 10″ done for 16 min per session two times per week for four weeks; each exercise was composed of eight levels with increased difficulty. (3) Results: Following vestibular and cognitive training, dyslexic children increased their reading speed; interestingly, such an increase persisted at least one month after training. (4) Conclusions: Vestibular and cognitive training could improve the vestibular network, which is well known for being involved in several cognition functions leading to reading improvement in dyslexic children. Adaptive mechanisms could be responsible for maintaining such improvement for at least one month.

## 1. Introduction

Dyslexia is a common learning difficulty that does not compromise oral or nonverbal reasoning skills, and it is reported in 5–10% of school-aged children [1]. In the literature, different theories have tried to explain the etiology of this disorder [2]. Phonological impairment is the most known hypothesis for dyslexia [3]; however, others researchers [4,5] suggested that dyslexia could be due to cerebellar impairments. Interestingly, reading performance and arithmetic skills have been suggested to also be related to cerebellar activity [6]. Several subsequent studies from our group [7,8] and others [9,10] examining postural performances in the dyslexia population supported this hypothesis, even if other researchers [11,12] suggested that such an impairment occurred not only in dyslexia but also in subjects having other types of developmental disorders. A visual attentional deficit has also been reported by Facoetti et al. [13] and our group showed an abnormal oculomotor pattern in children with dyslexia [14,15,16]. Based on these different theories, several researchers have tried to develop different training types in order to improve the reading abilities in children with dyslexia. 

In the literature, there are in fact several studies suggesting that visual attention and/or oculomotor training improve the reading skills in children with dyslexia [17,18]. In a more detailed way, Peters and collaborators [17] reviewed 18 studies reporting the beneficial effects of visual perceptual training on the reading skills in dyslexic subjects, leading to an improvement in reading fluency, comprehension and reading accuracy. Interesting, as reported by Meng et al. [19], the beneficial effects of a visual perceptual training on reading capabilities, such as reading fluency, in children with dyslexia could still be observed two months after the training. Our group [20] also observed that a short visual attentional training improved the reading speed in dyslexic children, shortening their duration of fixations. Solan and collaborators [21] reported that both traditional reading training as well as specific visual attention-based training improved the reading learning capabilities in dyslexic children, and a follow-up study of the same group [22] showed that an intensive visual attention training (12 one-hour sessions) increased reading comprehension in dyslexia children. These researchers advanced the hypothesis of a cognitive link between visual attention, oculomotor and reading skills, even if the exact neurophysiological relationship between these processes is still unknown.

Vander Stappen et al. [23] showed that rapid automatized training improved the reading performance in dyslexic children but also increased the activity of dorsal brain structures, particularly the left anterior segment of the arcuate fasciculus, which is well known for being related to oral language and reading activity. This finding suggested that rapid automatized naming training could lead to central adaptive mechanisms in the brain.

Beneficial effects have also been reported following working memory training in children with dyslexia, given that it well known that working memory abilities are poor in dyslexic children [24]. Yang et al. [25], for example, reported in Chinese children with dyslexia that the training of the verbal or visuospatial working memory could improve reading skills.

Importantly, we have to note that vestibular disabilities have been found to be related to various forms of cognitive impairments, such as visuospatial ability, attention, executive function, and memory [26,27]. Recently, our group [28] observed vestibular abnormalities in dyslexic children reported by deficits in the functional head vestibulo-ocular reflex. This reflex is specifically involved in the stabilization of the image on the retina during rapid movements of the head, and it plays an important role in the reading process [29]. These findings are in line with previous studies reporting that vestibular deficiencies could negatively affect sensory integrative functions such as balance, vision and reading in children [30].

Someone could ask the question of whether vestibular training could have a beneficial effect on reading performance in children with dyslexia, given that different types of vestibular training used in patients with vestibular loss improved not only the simple vestibular function (eliminating dizziness) but also executive functions like attention, visuo-spatial abilities and memory. Indeed, Sugaya et al. [31] tested whether a vestibular rehabilitation could improve both body stability and cognitive functions in a large group of adult subjects with dizziness. The vestibular rehabilitation program consisted of both head and eye exercises in a sitting or standing position, to develop strategies for improving the gaze stability by the cervico-ocular reflex. These authors reported a significant decrease of vertigo symptoms but also an improvement in the cognitive functions of these patients, supporting the evidence of the link between the vestibular system and cognition as suggested by the model by Ellis and colleagues [32].

In the present study, we aim to evaluate the effects of a short vestibular and cognitive training period on the reading performance in children with dyslexia. Based on previous cited studies we made the hypothesis that vestibular and cognitive training could have a beneficial effect on cognitive function such as reading speed in these children. 

## 2. Materials and Methods

### 2.1. Subjects

Nineteen children with dyslexia (11 females and 8 males) between the ages of 8.11 and 11 years (mean 9.50 ± 0.40) participated in the study. Children with dyslexia were recruited after a neuropediatric assessment; each child underwent an examination of reading and writing skills as well as phonological skills, visuo-attentional skills and verbal memory using a French battery (Batterie Analytique du Langage Ecrit, BALE [33]). This is the standard test often used in France for selecting a dyslexic population. The BALE test includes 40 subtests that cover a wide range of language and cognitive functions (oral language, reading, spelling, memory, phonological skills, visual processing). Inclusion criteria were: scores of BALE tests beyond 2 standard deviations; a normal means intelligence quotient (IQ, evaluated with WISC-IV; between 80 and 115); no known neurological or psychiatric abnormalities, no visual impairment or difficulty with near vision, and no hearing loss. Exclusion criteria were: any known neurological disorders, visual impairment, and any known vestibular disorder. One child was excluded from the study because of the presence of a visual deficit (microstrabismus). Based on the BALE scores on the reading test, all included dyslexic children had an abnormal reading speed with respect to their chronological age.

### 2.2. Study Design

**Figure 1:**
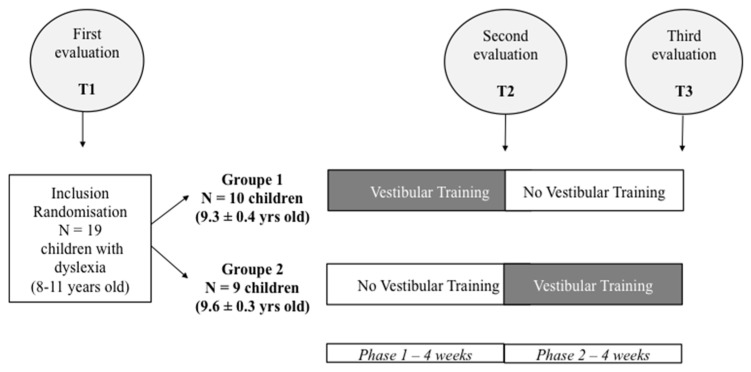
Illustration of the crossover interventional experimental design.

We conducted a crossover, interventional experimental design of a single-blind nature for the child (see Figure 1). In this design, each child acted as his or her own control, thus negating undesirable intersubject variability. Each child received both vestibular and cognitive training (VT) and no vestibular and cognitive training (no VT). 

During phase 1, every odd-numbered child first received VT, every even-numbered child first had no VT, and vice versa during phase 2. This interventional study had a duration of 8 weeks: it consisted of 4 weeks for each phase, 2 VT sessions/week, and each vestibular and cognitive training session lasted 16 min. During the no VT, the child did not perform any other training type to avoid the contamination of test results. 

### 2.3. Reading Task

The reading ability was evaluated for each child by the ELFE test (Évaluation de la Lecture en FluencE) (www.cognisciences.com, Grenoble, France). This test consisted of two texts, i.e., “Le Geant Egoiste” and “Monsieur Petit”. The child had to read one text aloud during 1 min, and the examiner counted the number of words read. Note that, at least in France, dyslexic children are invited to read aloud instead of reading silently, allowing the therapists to correct reading errors. It should be noted that the two texts are similar and comparable in terms of their difficulty and orthography. In order to avoid any risk of the learning effect, French clinicians frequently use these texts to measure the reading capability before and after training. Note that all dyslexic children tested at T1 had an abnormal reading speed in the ELFE test (this is one of the inclusion criteria). Indeed, the number of words read in 1 min by nondyslexic children from 8 to 11 years old is between 95–141 words/min. As shown in Table 1, all dyslexic children had a slow reading speed.

### 2.4. Vestibular and Cognitive Training Procedure

**Figure 2:**
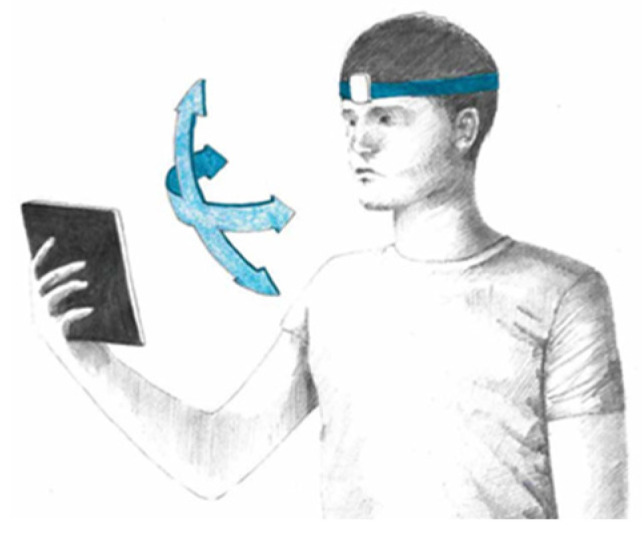
Experimental set-up used for *Gym* and *Read* exercises.

For the vestibular and cognitive training, we used a system developed by BeonSolution S.r.l. (patent: BS-0002-EP-ORD); it consisted in four different exercises done for 16 min per session 2 times per week for 4 weeks. In order to maximize the training effect, each exercise was composed of 8 levels with increased difficulty. A speech therapist was always present during the session and supervised it. The four exercises (*Gym, Read, Touch and Memory*) are part of the software Functional Head Impulse Test (FHIT R2) and were presented on a Wacom tablet 10″. The child was seated on a comfortable chair and performed the test by holding the tablet with his hands. During the training time, for two of the four exercises only (*Gym and Read*), the child had a head-mounted gyroscope to measure the head angular velocities in order to be sure that the head velocity reached optimal values (Figure 2). In other words, during these two exercises, the child had to move its head to stimulate vestibular compensation and improve VOR capabilities. 

To perform the other two exercises (*Touch and Memory*), the child did not need any head movement detection. The four exercises were randomly presented to the child.

**Figure 3:**
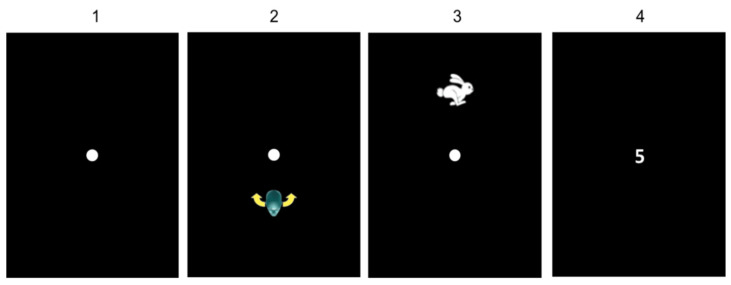
*Gym* exercise.

In the *Gym* exercise, the child had to rotate his head to the right, left, up or down randomly, as requested by the program. The child fixed a central dot (1 cm), and afterwards he moved his head initially at 80°/s (Figure 3(1)). If the head movement was not sufficiently wide, a head with arrows appeared to explain to the child the type of movement that needed to be done (Figure 3(2)). If the head movement was too fast, a hare appeared on the screen indicating to the child that he had to move his head slowly (Figure 3(3)). In both cases, the child had to do a head movement until he reached the corrected head speed. When the head movement was correctly done, a number (1 cm) appeared in the middle of the screen for 120 ms (Figure 3(4)). Afterwards, the child was invited to select the number seen on the list of numbers seen on the bottom of the tablet; the child had 10 s to answer. As the level increased, the head velocity increased (from 80 deg/s to 150 deg/s).

In the *Read* exercise, the child had to make rotations of the head to the right and left on the longitudinal body axis only. The program was similar to those of the Gym exercise, but instead of a simple number the child had to read a word of 3 letters (0.5 cm each) that appeared on the screen for 200 ms. The child had to remember the sequence of letters and select the same sequence seen among five that were displayed on the bottom of the tablet. As the level increased, the number of letters increased. 

**Figure 4:**
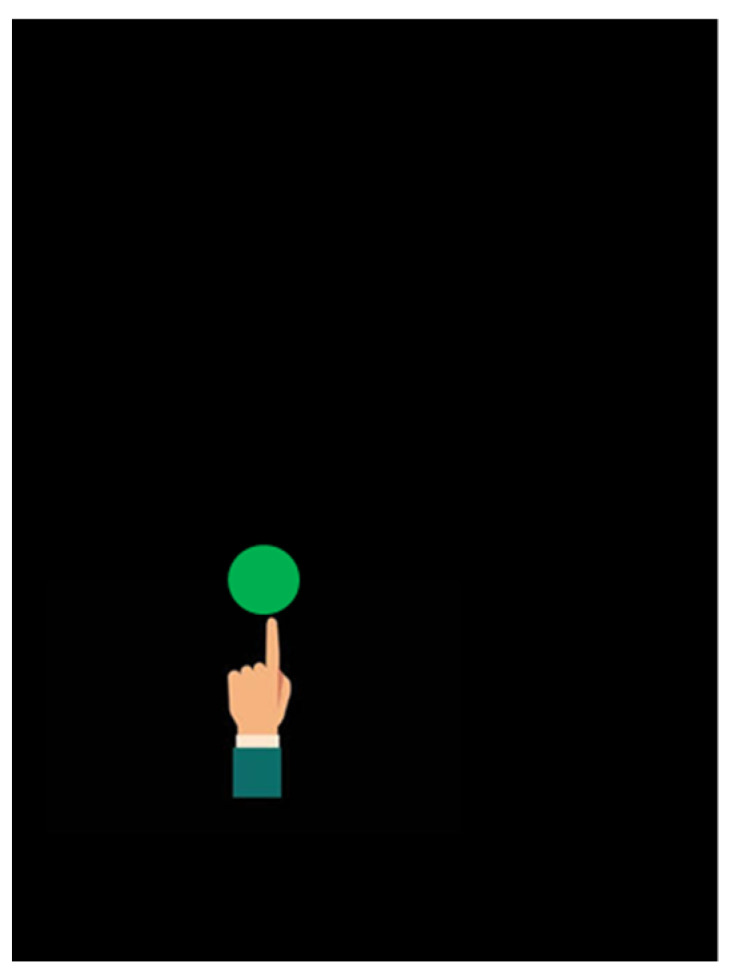
*Touch* exercise.

In the *Touch* exercise, the child had to respond to a visual stimulus as soon as possible. A green dot 1 cm in diameter appeared randomly on the screen (black background) for a time period of 1 s. The child was asked to touch it with his finger before it disappeared (see Figure 4).

The child had to touch the highest number of dots in the set time. As the level increased, the time during which the dot appeared decreased (from 1 s to 400 ms).

**Figure 5:**
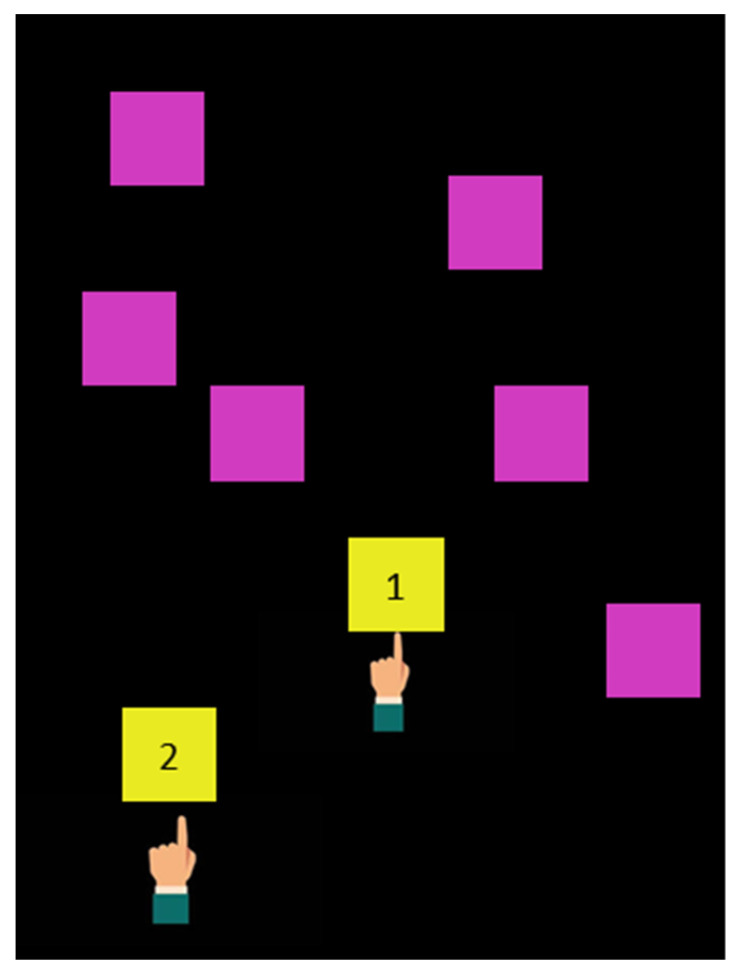
*Memory* exercise.

The *Memory* exercise aims to train the spatial memory of the child. The child was asked to observe a sequence of purple square stimuli (2.7 × 2.7 cm) appearing randomly for 500 ms on the screen one stimulus at a time. The child was invited to touch each stimulus with his finger, changing its color from purple to yellow (see Figure 5). 

The exercise consisted in remembering the sequence of the stimuli and repeating it on the screen. At the beginning of the session, only 2 stimuli appeared on the screen. Afterwards, if the child’s response was correct, the number of stimuli increased up to nine. 

In sum, this training type was based on vestibular and visual exercises (*Gym* and *Read*) and on attentional and memory exercises (*Touch* and *Memory*).

Note that the difficulty of each of these exercises increased when the child performed 90% of the exercise correctly.

### 2.5. Data Analysis

The number of words read during 1 min in the ELFE test was the variable that was compared in the three different periods (baseline, after VT and no VT).

### 2.6. Statistical Analysis

Statistical analysis was performed with the Statistica software (STATISTICA^®^ (12.0, Palo Alto, CA, USA). A repeated-measures, one-way analysis of variance was used on the ELFE test measured in baseline, after VT and no VT; post-hoc Bonferroni analyses were performed. In order to explore the eventual lasting vestibular and cognitive training effect, for each group of dyslexic children the paired t-test was used to compare the ELFE test measures at three different times (in T1 versus T2, in T2 versus T3, and in T1 versus T3). The effect of a factor was considered as significant when the *p*-value was below 0.05. 

## 3. Results

**Figure 6:**
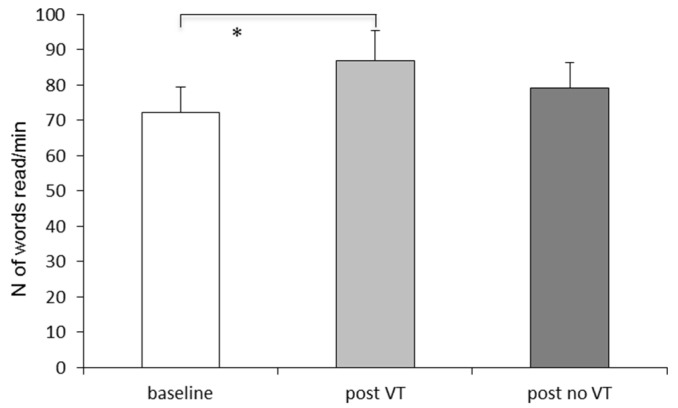
Mean and standard error of the number of words read in 1 min in the three different periods (baseline, post VT and post no VT). Asterisk indicates significant difference between the two conditions.

The number of words read in 1 min in the three different periods is reported in Figure 6. We observed a significant effect of the period (F_(2,36)_ = 10.70, *p* < 0.0002). The Bonferroni post-hoc test reported that, with respect to the baseline, the number of words read in 1 min increased significantly after the vestibular and cognitive training only (post VT, *p* < 0.0001).

In order to explore the eventual lasting vestibular and cognitive training effect, in each group of children with dyslexia the number of words read in 1 min was compared by using the *t*-test in the three different times (T1, T2 and T3, see Table 1). 

For the group G1 who performed the vestibular and cognitive training at T2, the *t*-test reported a significant difference between T1 versus T2 (t = 4.88, *p* < 0.0008) and between T1 versus T3 (t = 3.89, *p* < 0.003); in contrast, the t-test failed to show a significant difference between T2 versus T3 (t = 0.41, *p* = 0.68). For the group G2 who performed the vestibular and cognitive training at T3, the t-test reported a significant difference between T1 versus T3 (t = 2.49, *p* < 0.03) and between T2 versus T3 (t = 4.78, *p* < 0.001), while the difference between T1 and T2 did not reach significance (t = 0.58, *p* = 0.58). Note, however, that 6/19 dyslexic children only reached a normal reading speed after vestibular and cognitive training (see Table S1 in the supplementary information). 

## 4. Discussion

The main finding of this study is that a short vestibular and cognitive training period affects the reading speed in children with dyslexia, leading them to read more rapidly. Interestingly, such an improvement persists at least four weeks after the end of this training type. These findings are discussed below. 

The different exercises of the vestibular and cognitive training used in the present study were developed in order to improve specific activities essential for the reading task. In order to reach a good reading performance, children have to stabilize their eyes on the text to read, and at the same time the visual and spatial attention as well as memory abilities need to work together and in a correct way in order to understand the text to be read. Indeed, these functions are fundamental to reading efficiently and are used by the child to connect letters and graphemes with the corresponding sounds and to understand the meaning of all words/sentences [34]. Several studies reported that in dyslexic children all these components were deficient; for instance, a poor gaze stabilization [35,36,37], impaired visual attention [38] and poor working memory [39] have been reported, and distinct training types reinforcing each of these abilities have been found to be efficient in order to improve the reading skills in dyslexic children (see Introduction).

The novelty of the training used in the present study is that we decided to reinforce the vestibular system together with all of the functions cited above. This study, even if is based on the reading speed only, suggested that this vestibular and cognitive training type on a tablet could be useful for dyslexic children in order to improve their reading performance. Note, however, that further studies will be necessary to explore whether this vestibular and cognitive training type could improve the reading and comprehension capabilities in children with dyslexia. 

The finding of the present study also allows one to point out the role of the vestibular system in reading and more generally in cognitive capabilities. Indeed, even if the vestibular system has always been thought of as being important for balance control, being particularly responsible for head acceleration and generating vestibulo-ocular reflexes to stabilize the visual image on the retina [40], it also important for cognition, vision and perception [30,41,42], and several studies have already reported poor cognitive functions in subjects with vestibular deficiencies [43,44]. The working mechanism responsible for such an improvement cannot be described by the present behavioral study; however, the dorsal pathway has for a long time been considered important for attention and visual word recognition [45]. Further neurophysiological studies are needed to gain more insight into the cortical structures in charge of such changes. 

Another finding of this study is that improvement in the reading speed persisted at least one month after training, suggesting that adaptive mechanisms could be responsible for such improvement, even if this hypothesis needs to be confirmed with MRI studies. This is an interesting result having important consequences in patient care, given that clinicians could use this vestibular and cognitive training for the rehabilitation of the reading speed in dyslexic children.

Future clinical trials conducted over a longer time period will be necessary to investigate whether the benefit of such vestibular and cognitive training is maintained beyond one month and also to evaluate whether dyslexic children reach a normal reading speed. An eye-tracker study will also allow one, after training, to evaluate eventual changes in the oculomotor pattern in these children. 

One could also ask the question of whether only vestibular training could improve the reading performance in dyslexic children. Based on previous studies describing patients with vestibular deficits, we could make the hypothesis that dyslexic children could benefit from Gym and Read exercises; most likely a longer and more frequent training period in dyslexic children will be necessary to test such a hypothesis. The choice of training both vestibular and cognitive functions was done based on our experience and conviction that only one type of training is not useful enough for helping dyslexic children improve their reading difficulties. Indeed, dyslexia is a multifactorial deficit requiring several competences in order to be overcome [2].

Finally, in this study we did not distinguish between different types of dyslexia [46,47]. Vestibular and cognitive training could have been more efficient in dyslexics who had visual-attentional deficits rather than a phonological deficit, given that the trained processes were more related to visuo/spatial attentional activities than to phonological skills. 

## 5. Conclusions

A short vestibular and cognitive training period based on four exercises (based on visual and spatial attention, and on memory abilities) made on a tablet is able to increase the reading speed in children with dyslexia. Importantly, it seems that such an improvement persists at least one month afterwards. This type of training could be an easy and practical tool to improve the reading performance in dyslexic children.

## 6. Patents

BeonSolutions S.r.l. has applied for a patent for the technology used to conduct vestibular training (Device and method for the prevention and treatment of dyslexia, EP21150962 & EP21150962.5).

## Figures and Tables

**Table 1:** Mean, standard error of the number of words read in 1 min for the two groups of children with dyslexia tested in the three different time periods. * Indicates a significant difference with respect to T1, while # indicates a significant difference with respect to T3.

	T1	T2	T3
G1	54 ± 10	69 ± 11 *	69 ± 11 *
G2	92 ± 4	89 ± 6 **#**	107 ± 8 *

## Data Availability

The datasets analyzed during the current study are available from the corresponding author on reasonable request.

## Supplementary Materials

The following are available online at https://www.mdpi.com/article/10.3390/brainsci11111440/s1, Table S1: Age (years) and number of words read in 1 min from each child tested three times (at T1, T2 and T3).
