# Supplementary material for: Short Vestibular and Cognitive Training Improves Oral Reading Fluency in Children with Dyslexia
Source: Brain Sci. 2021 Oct 29;11(11):1440. doi: 10.3390/brainsci11111440 (PMC8615463; doi:10.3390/brainsci11111440)
Supplement: Supplementary file 1 [file brainsci-11-01440-s001.zip › brainsci-1420172-supplementary.pdf]

Table S1: Age (years) and number of words read in 1 min from each child tested three times (at T1, T2 and T3).

G1:

Age	T1	T2	T3
9.1	61	73	78
9.11	24	31	28
9	88	91	91
9.11	21	37	39
8.6	25	37	55
9.2	52	70	71
9.4	30	35	28
10	94	113	103
9.11	40	57	58
11	108	144	147

G2:

Age	T1	T2	T3
9.7	92	84	92
11	91	86	95
8	70	70	74
9.7	86	86	102
9.7	90	76	102
10.6	101	82	99
11	110	123	162
9.3	98	115	137
8.11	80	85	98
